# A case of rectal MiNEN demonstrating vascular stalk-like imaging and gross demarcation of tumor components

**DOI:** 10.1093/jscr/rjaf996

**Published:** 2025-12-18

**Authors:** Masato Hayashi, Masanori Kotake, Hiroki Kitabayashi, Kazuki Kato, Daisuke Fujimori, Takahiro Yoshimura, Koichiro Sawada, Hironori Hayashi, Kaeko Oyama, Takuo Hara, Kenichiro Tsukada, Munenori Mukai, Noriyuki Inaki

**Affiliations:** Department of Surgery, Koseiren Takaoka Hospital, Eirakumachi 5-10, Takaoka, Toyama 933-8555, Japan; Department of Surgery, Koseiren Takaoka Hospital, Eirakumachi 5-10, Takaoka, Toyama 933-8555, Japan; Department of Surgery, Koseiren Takaoka Hospital, Eirakumachi 5-10, Takaoka, Toyama 933-8555, Japan; Department of Surgery, Koseiren Takaoka Hospital, Eirakumachi 5-10, Takaoka, Toyama 933-8555, Japan; Department of Surgery, Koseiren Takaoka Hospital, Eirakumachi 5-10, Takaoka, Toyama 933-8555, Japan; Department of Surgery, Koseiren Takaoka Hospital, Eirakumachi 5-10, Takaoka, Toyama 933-8555, Japan; Department of Surgery, Koseiren Takaoka Hospital, Eirakumachi 5-10, Takaoka, Toyama 933-8555, Japan; Department of Surgery, Koseiren Takaoka Hospital, Eirakumachi 5-10, Takaoka, Toyama 933-8555, Japan; Department of Surgery, Koseiren Takaoka Hospital, Eirakumachi 5-10, Takaoka, Toyama 933-8555, Japan; Department of Surgery, Koseiren Takaoka Hospital, Eirakumachi 5-10, Takaoka, Toyama 933-8555, Japan; Department of Medical Oncology, Koseiren Takaoka Hospital, Eirakumachi 5-10, Takaoka, Toyama 933-8555, Japan; Department of Pathology, Koseiren Takaoka Hospital, Eirakumachi 5-10, Takaoka, Toyama 933-8555, Japan; Department of Gastrointestinal Surgery, Breast Surgery, Kanazawa University Graduate School of Medical Sciences, 13-1 Takaramachi, Kanazawa, Ishikawa 920-8640, Japan

**Keywords:** rectal cancer, MiNEN, vascular stalk-like structure, robot-assisted surgery

## Abstract

Mixed neuroendocrine–non-neuroendocrine neoplasms (MiNENs) of the rectum are extremely rare and pose significant diagnostic challenges. We report a 69-year-old man with rectal MiNEN successfully treated with a robot-assisted Hartmann’s procedure. Preoperative contrast-enhanced CT revealed a vascular stalk-like structure within the tumor. Gross pathology showed a clear demarcation between adenocarcinoma and small-cell neuroendocrine carcinoma. Robot-assisted surgery facilitated precise dissection in the narrow pelvic cavity and safe distal transection through a transanal approach. This case highlights unique radiological and pathological findings that may aid in the diagnosis and management of rectal MiNEN.

## Introduction

Mixed neuroendocrine–non-neuroendocrine neoplasms (MiNENs) were first formally defined in the 2010 World Health Organization (WHO) classification and subsequently revised in the 2019 edition [1]. According to the WHO, MiNENs are biphasic epithelial malignancies comprising both neuroendocrine and non-neuroendocrine components, with each component constituting at least 30% of the tumor [2]. They most commonly arise in the stomach or colon but are exceedingly rare in the rectum. Due to their dual histology and nonspecific imaging, accurate preoperative diagnosis is often difficult, and standardized treatment strategies remain undefined. We present a case of rectal MiNEN with distinctive imaging and pathological findings managed by robot-assisted surgery.

## Case report

**Figure 1:**
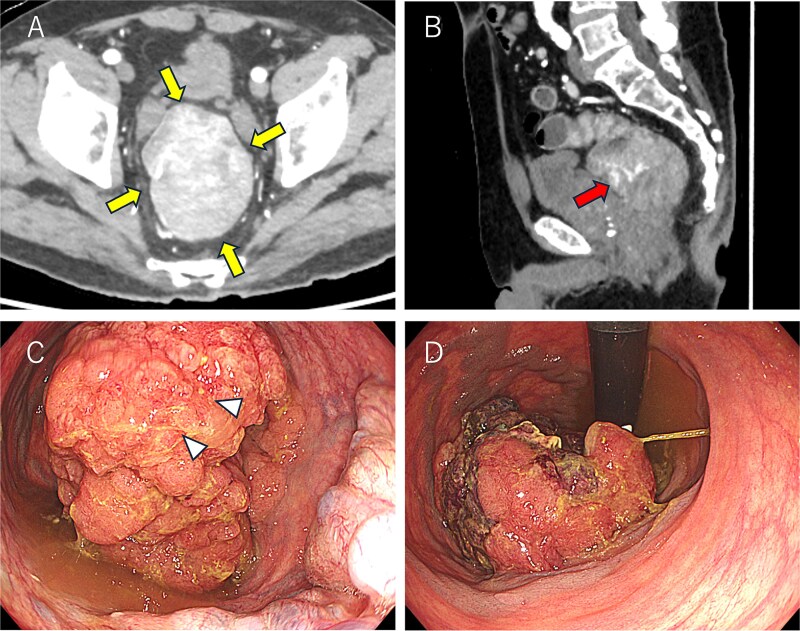
Colonoscopy and computed tomography findings. (A) A mass measuring 8.5 × 6.5 cm was observed in the lower rectum (arrow). (B) A vascular stalk-like structure was noted in the proximal portion of the tumor (arrow). (C, D) A large mass occupying the rectal lumen was observed 3 cm from the anal verge (D: retroflexed endoscopic view). The arrowhead indicates the biopsy site.

A 69-year-old man presented with diarrhea and anemia. Contrast-enhanced CT revealed an 8.4 × 6.3 cm rectal mass with a vascular stalk-like structure, with the proximal portion appearing particularly vascular and a single enlarged lymph node (Fig. 1A and B). Colonoscopy identified a large tumor 3 cm from the anal verge (Fig. 1C and D), and biopsy showed high-grade adenoma; carcinoma could not be excluded. A robot-assisted Hartmann’s procedure was performed. Intraoperatively, the bulky tumor filled the pelvic cavity, making manipulation difficult, but articulating robotic instruments allowed precise distal dissection. Due to limited pelvic space, transabdominal stapling was not feasible; rectal transection was completed transanally. The operative time was 480 minutes, with minimal blood loss. Postoperative recovery was uneventful. The patient is currently undergoing capecitabine plus oxaliplatin therapy as adjuvant chemotherapy, and no signs of recurrence or metastasis were found 3 months after surgery.

**Figure 2:**
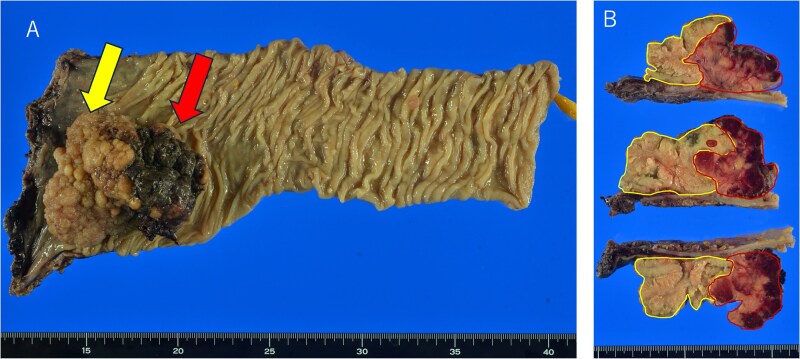
Macroscopic findings of the resected specimen. (A) A tumor measuring 8.5 × 7.0 cm protrudes into the intestinal lumen, with surface necrosis observed on the oral side (red arrow) and multiple elevated lesions on the anal side (yellow arrow). (B) Sectional images reveal a clear demarcation between the two components (red lines: neuroendocrine carcinoma; yellow lines: adenocarcinoma).

**Figure 3:**
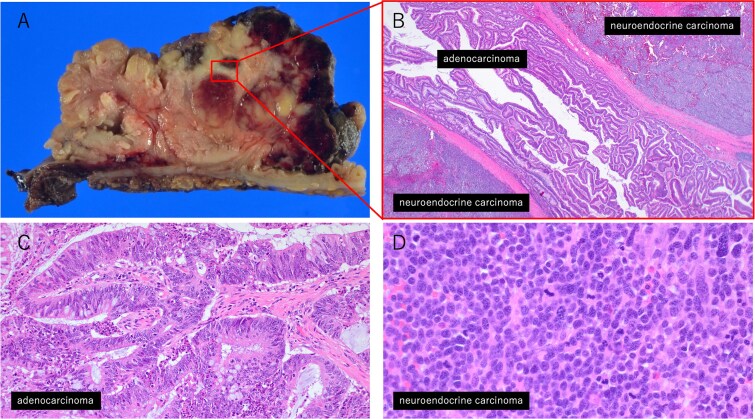
Histopathological findings of the tumor. (A) Macroscopic view of the tumor. (B) Low-magnification image (×20) showing the interface between the adenocarcinoma and neuroendocrine carcinoma components. (C) High-magnification image (×200) of the adenocarcinoma area. (D) High-magnification image (×400) of the neuroendocrine carcinoma area.

**Figure 4:**
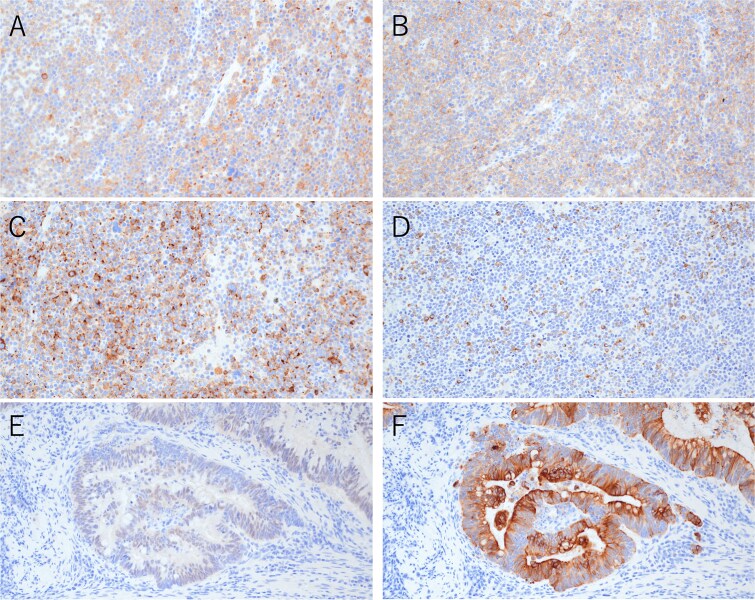
Immunohistochemical findings of the primary tumor (×200). (A) Synaptophysin staining in neuroendocrine carcinoma. (B) CD56 staining in neuroendocrine carcinoma. (C) Chromogranin A staining in neuroendocrine carcinoma. (D) CAM5.2 staining in neuroendocrine carcinoma. (E) CDX2 staining in adenocarcinoma. (F) CK20 staining in adenocarcinoma.

Postoperative pathological examination revealed that the tumor measured 8.5 × 7.0 cm and was invading the muscularis propria (Fig. 2A). Metastases were detected in three of the 18 regional lymph nodes removed. Histological examination revealed that the tumor comprised ~50% moderately differentiated adenocarcinoma and 50% small cell neuroendocrine carcinoma (Fig. 3A–D). Immunohistochemically, the neuroendocrine component was positive for synaptophysin and CD56, while CAM5.2 and chromogranin A were focally positive (Fig. 4A–D). The adenocarcinoma component stained positive for CK20 and CDX2, indicating its origin from the colorectal epithelium (Fig. 4E and F). Gross examination of the surgical specimen revealed a clear macroscopic demarcation between the two components (Fig. 2B). The neuroendocrine region appeared solid and reddish brown, whereas the adenocarcinoma component appeared solid and yellowish brown.

**Figure 5:**
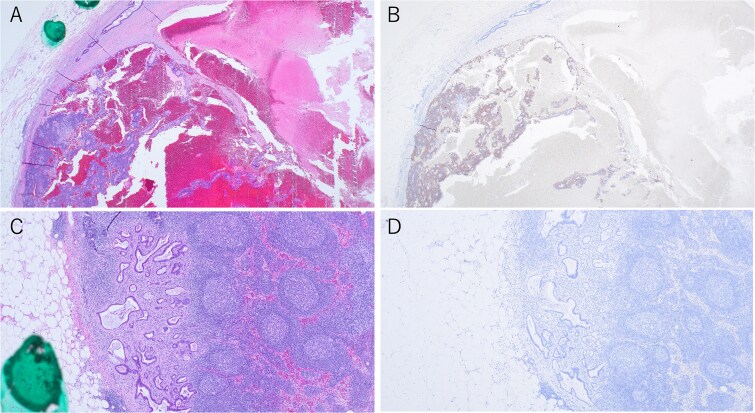
Immunohistochemical findings of metastatic lymph nodes. (A, B) Lymph node metastases showing both tubular adenocarcinoma and neuroendocrine carcinoma components, with synaptophysin staining (×40). (C, D) Lymph node metastases of tubular adenocarcinoma, with synaptophysin staining (×40).

Metastatic involvement was observed in 3 of the 18 regional lymph nodes. One of the metastatic lesions was positive for synaptophysin and contained both tubular adenocarcinoma and neuroendocrine carcinoma (Fig. 5A and B). The remaining two lymph nodes contained only tubular adenocarcinoma (Fig. 5C and D).

## Discussion

Rectal MiNENs are rare entities that often present diagnostic difficulties, as imaging is typically nonspecific and biopsy may not represent both components. The prognosis is generally determined by the more malignant component, often the neuroendocrine carcinoma, which tends to be highly proliferative and associated with early metastasis [3, 4]. In our case, CT demonstrated a vascular stalk-like structure within the tumor, which may correspond to the hypervascular nature of the neuroendocrine component. Such imaging characteristics, in conjunction with the clinical and endoscopic features, may raise the suspicion of mixed histology and warrant further pathological investigation [5]. Furthermore, gross examination revealed a striking macroscopic demarcation between adenocarcinoma and neuroendocrine carcinoma, a feature rarely documented in MiNENs. Such findings provide valuable diagnostic clues and may help suspect mixed histology preoperatively.

## Conclusions

This case demonstrates unique imaging and pathological features of rectal MiNENs and emphasizes the importance of considering this rare diagnosis when atypical findings are encountered. Robot-assisted surgery may be a useful option for safe resection in anatomically challenging cases.

## Data Availability

Data sharing is not applicable to this article because datasets were neither generated nor analyzed for the case report.
